# Does latissimus dorsi tendon transfer provide a normal scapular rhythm and external rotation strength in posterosuperior massive irreparable rotator cuff tears? A kinematic analysis in a retrospective cohort

**DOI:** 10.1186/s10195-026-00910-0

**Published:** 2026-03-04

**Authors:** Cristobal Calvo, Gabriele Fiumana, Alberto Brigo, Elena Dora Ruggiero, Rocco Bonfatti, Alessandro Donà, Gian Mario Micheloni, Andrea Giorgini, Luigi Tarallo, Giuseppe Porcellini

**Affiliations:** 1 Facultad de Medicina, Universidad de los Andes, Santiago, Chile; 2 Hospital Militar de Santiago, La Reina, Chile; 3 Shoulder and elbow team, Forlì, FC Italy; 4 Orthopaedics and Trauma Unit, University of Modena and Reggio Emilia, Modena, Italy

**Keywords:** Latissimus dorsi tendon transfer, Massive irreparable rotator cuff tear, Scapulothoracic rhythm, Shoulder biomechanics, Kinematic analysis

## Abstract

**Background:**

Posterosuperior massive irreparable rotator cuff tears (PMIRT) are rare and disabling conditions. When conservative treatment fails, latissimus dorsi tendon transfer (LDTT) is a viable surgical option for symptom relief in carefully selected patients. However, its effectiveness in restoring glenohumeral function and its influence on scapulothoracic rhythm remain subjects of ongoing debate.

**Purpose:**

The purpose of this study was to evaluate the clinical outcomes of LDTT and assess its impact on scapulothoracic rhythm using kinematic and electromyographic analysis.

**Material and methods:**

A total of 18 patients with PMIRT underwent LDTT. Functional scores consisted of Constant-Murley score (CMS), America Shoulder and Elbow Surgeons (ASES) score, and Quick Disabilities of the Arm, Shoulder, and Hand (QuickDASH) score. Electromyography (EMG) activity of the latissimus dorsi muscle was evaluated in conjunction with three-dimensional (3D) kinematic tracking system. Differences in external rotation strength were compared with and without active adduction.

**Results:**

The mean age was 55.9 years (range 40–69 years), with a mean follow-up of 20.4 months (range 6–39 months). At final follow-up, the mean CMS was 68.2 (95% CI 64.9–71.5), ASES was 76.9 (95% CI 66.1–87.6), and QuickDASH was 17.6 (95% CI 9.5–25.6). A significant difference in external rotation strength was observed with and without active adduction (*p* < .0005). EMG confirmed latissimus dorsi activation in all patients, with no significant differences between rotation conditions (*p* > .05). Kinematic analysis showed an overall normal scapulothoracic rhythm, with significant differences only in scapular tilting during elevation and external rotation with the shoulder in adduction (*p* = 0.044 and *p* = 0.023, respectively).

**Conclusions:**

LDTT provides satisfactory clinical outcomes in patients with PMIRT, enhancing external rotation strength when the latissimus dorsi is actively recruited and contributing to near-normal scapulothoracic rhythm restoration.

A structured, targeted postoperative rehabilitation protocol is essential to optimize outcomes.

**Level of evidence:**

IV.

## Introduction

Posterosuperior massive irreparable rotator cuff tear (PMIRT) is a disabling condition of the shoulder characterized by superior migration of the humeral head and tears of the rotator cuff tendons, with significant atrophy of the supraspinatus and infraspinatus muscles—greater than 60% of muscle belly as classified by Thomazeau [19]. Patients commonly report shoulder pain, weakness, and limited function in forward flexion and external rotation. When conservative treatment fails, surgical options include partial repair, superior capsular reconstruction, subacromial balloon spacer, reverse shoulder arthroplasty (RSA), and tendon transfers [4, 16, 17].

Latissimus dorsi tendon transfer (LDTT) is a well-established procedure for managing PMIRT in patients with intact subscapularis and teres minor tendons and no significant glenohumeral joint degeneration [2–5, 8, 12, 15]. However, its precise role remains controversial. Some studies suggest LDTT primarily acts as a humeral depressor via tenodesis, while others propose an active contribution to external rotation [4]. Latissimus dorsi activity in external rotation movements after LDTT is still debated [11]. The post-transfer activity of the latissimus dorsi in external rotation remains debated. Furthermore, despite growing recognition of scapulothoracic rhythm in shoulder function, its relationship with LDTT remains poorly understood [7, 13].

The aim of our study was to (1) evaluate the contribution of LDTT on scapulothoracic rhythm, (2) assess LD activation post-transfer, (3) determine its contribution to external rotation strength, and (4) analyze clinical outcomes following LDTT.

## Material and methods

This retrospective study included patients who underwent LDTT between July 2020 and June 2023 at our institution. Inclusion criteria were: (1) Thomazeau grade III of supraspinatus and infraspinatus muscle atrophy [19], (2) failure of conservative treatment for at least 6 months, (3) a healthy contralateral shoulder, and (4) a minimum follow-up of 6 months postoperatively. Exclusion criteria were: (1) radiographic evidence of glenohumeral osteoarthritis and (2) magnetic resonance imaging (MRI) evidence of subscapularis or teres minor tear.

A total of 26 patients were eligible; 4 were lost to follow-up, 3 declined to participate, and 1 underwent RSA due to the unsatisfactory results after LDTT. Therefore, 18 patients were included. Preoperative evaluation involved both a surgeon and a physical therapist, focusing on functional limitations, occupational demands, and pain assessment.

### Surgical technique

Patients were positioned in lateral decubitus. The latissimus dorsi (LD) tendon was harvested via a posterior axillary approach, preserving the neurovascular bundle. The tendon was prepared with Krakow sutures and mobilized to reach the greater tuberosity. Arthroscopic biceps tenotomy was performed, and the tuberosity was prepared for fixation. The LD tendon was passed between the deltoid and teres minor and fixed using two PopLock knotless anchors (ConMed, Largo, FL, USA) in a double-row configuration on the posterosuperior tuberosity. One knotless anchor was placed at the level of the greater tuberosity (posterior and lateral to the bicipital groove), while the other was positioned laterally to the greater tuberosity to avoid the boutonnière effect (interposition of the graft between the humerus and the glenoid).

### Postoperative care

For the first 6 weeks, the shoulder was immobilized using a 15° abduction and external rotation pillow. Passive elbow and hand motion was permitted immediately. Gentle passive elevation in the scapular plane was initiated on postoperative day 21 to minimize the risk of postoperative stiffness.

From day 40 onward, active-assisted movements and self-stretching exercises in water, together with passive mobilization in all planes, were typically initiated in the outpatient setting to facilitate recovery of range of motion. From day 75, exercises using elastic resistance bands and light weights were introduced, with clear instructions regarding exercise execution and dosage.

Hydrokinesiotherapy involved the use of flotation devices to actively reinforce humeral head depressor muscles and strengthen the scapular pivot muscles. Hydrodynamic resistance tools (such as paddles) were employed to strengthen the internal and external rotator muscles, with particular emphasis on restoring external rotation.

Following this procedure, the original motor pattern typically resulted in preferential activation of the infraspinatus muscle rather than the latissimus dorsi, without producing effective movement. To promote the development of a new motor pattern, neuromuscular retraining was emphasized. Motor facilitation strategies included placing towels or pillows between the arm and trunk and using flotation devices during hydrotherapy. Patients were instructed to adduct the arm while performing external rotation to facilitate recruitment of the latissimus dorsi.

### Clinical evaluation

Demographic and clinical data were recorded. Physical examination included range of motion and specific rotator cuff test (Jobe test, resisted external rotation, belly press test, lift-off test).

A digital traction dynamometer measured strength in scapular plane elevation and external rotation with no abduction. External rotation strength was assessed with the arm adducted at rest (ERF) and during active adduction against a pillow (ERADF). Results were compared with the contralateral shoulder. Functional outcomes were assessed using Constant-Murley (CMS) score, Assessment Shoulder and Elbow Scale (ASES), and Quick Disability of the Arm, Shoulder and Hand (QuickDASH). Tendon integrity was evaluated via ultrasound at 6 months.

### Scapulothoracic rhythm and electromyography

Scapular kinematics were assessed dynamically using the ShowMotion™ system (NCS, Carpi, Italy), which combines kinematic and electromyography (EMG) sensors. Kinematic sensors were placed on the sternum, scapular spine, upper arm, and forearm. Surface EMG electrodes were applied over the latissimus dorsi belly. The system provided real-time movement and EMG data (microvolts). As surface EMG lacks standardization and it does not provide a reproducible measurement between patients, we analyzed the percentage of intra-patient change in EMG activity and strength between ERF and ERADF to evaluate latissimus dorsi contribution.

### Statistical analysis

Normality was tested using the Wilcoxon test. Student’s *t*-tests and Mann–Whitney *U* tests were applied as appropriate. Significance was set at *p* < 0.05.

## Results

A total of 18 patients (15 male, 3 female) were included (Table 1). Mean age at surgery was 55.9 years (range 40–69 years), and mean follow-up was 20.4 months (range 6–39 months). The right shoulder was affected in (88.9%) of cases. No intraoperative or postoperative complications were recorded. All tendon transfers remained intact at 6-month ultrasound evaluation.

**Table 1:** Sample characteristics

*n*	18	
Sex	83.3% (15) male	
Dominant arm	88.9% (16) right-handed	
Operated side	88.9% (16) right	
Operated dominant side	77.8% (14)	
Age at surgery (years)	55.9	(range 40–69)
Age at follow-up (years)	58.1	(range 43–71)
Follow up (months)	20.4	(range 6–39)

At the final follow up, patients demonstrated good ROM and functional scores (Table 2). Jobe test remained positive in 15 patients (83.3%). Resisted external rotation was positive in nine (50%) patients, and two patients (11.1%) had persistent lag sign.

**Table 2:** Clinical outcomes

	Operated side		Healthy side		
	Mean	SD	Mean	SD	*p*
Range of movements (degrees)					
Elevation	167.9	5.9	168.2	5.3	0.79
Abduction	174.9	6.7	177.8	4.2	0.05
External rotation	48.4	18.3	59.1	10.9	0.007

	Mean	95% CI	Mean	95% CI	*p*
Strength (kg)					
Elevation	3.5	2.53–4.47	7.94	6.75–9.12	< .0001
ERF	5.47	4.11–6.84	9.47	8.08–10.85	0.0001
ERADF	7.39	5.88–8.91	9.64	7.96–11.32	0.0436
ERADF-ERF difference	1.92	0.97–2.87	0.17	− 0.58–0.93	0.0049
EMG*					
EMG activity difference *(% ERADF over ERF)*	26.02	12.91–39.13	14.93	4.24–25.63	0.178

*ERF* external rotation force, *ERADF* external rotation + adduction (LD activation) force, *EMG* electromyography

^*^EMG is expressed as a percentage of intra-patient change because surface EMG does not provide a standardized and reproducible measurement between patients

Mean CMS on the operated side was 68.2 (95% CI 64.9–71.5), compared with 81.8 (95% CI 78.4–85.3) on the contralateral side (*p* < 0.001). Meanwhile ASES and QuickDASH scores were 76.9 (95% CI 66.1–87.6) and 17.6 (95% CI 9.5–25.6), respectively.

Strength on the healthy side was significantly greater than on the operated side in elevation and external rotation, both with (ERADF) and without (ERF) voluntary activation of LD (Table 2). A significant difference was found between ERF and ERADF on the operated side (*p* = 0.0005), but not on the healthy side (*p* = 0.636). The inter-shoulder comparison of ERADF–ERF difference also reached significance (*p* = 0.0049). External rotation ROM differed by 10.7° between sides in the neutral position (*p* = 0.007).

All patients showed latissimus dorsi activation during elevation and external rotation. EMG activity increased during ERADF compared with ERF, with a mean increase of 26.85% on the operated side and 14.93% on the healthy side, yet it was not significant (*p* = 0.178).

**Fig. 1:**
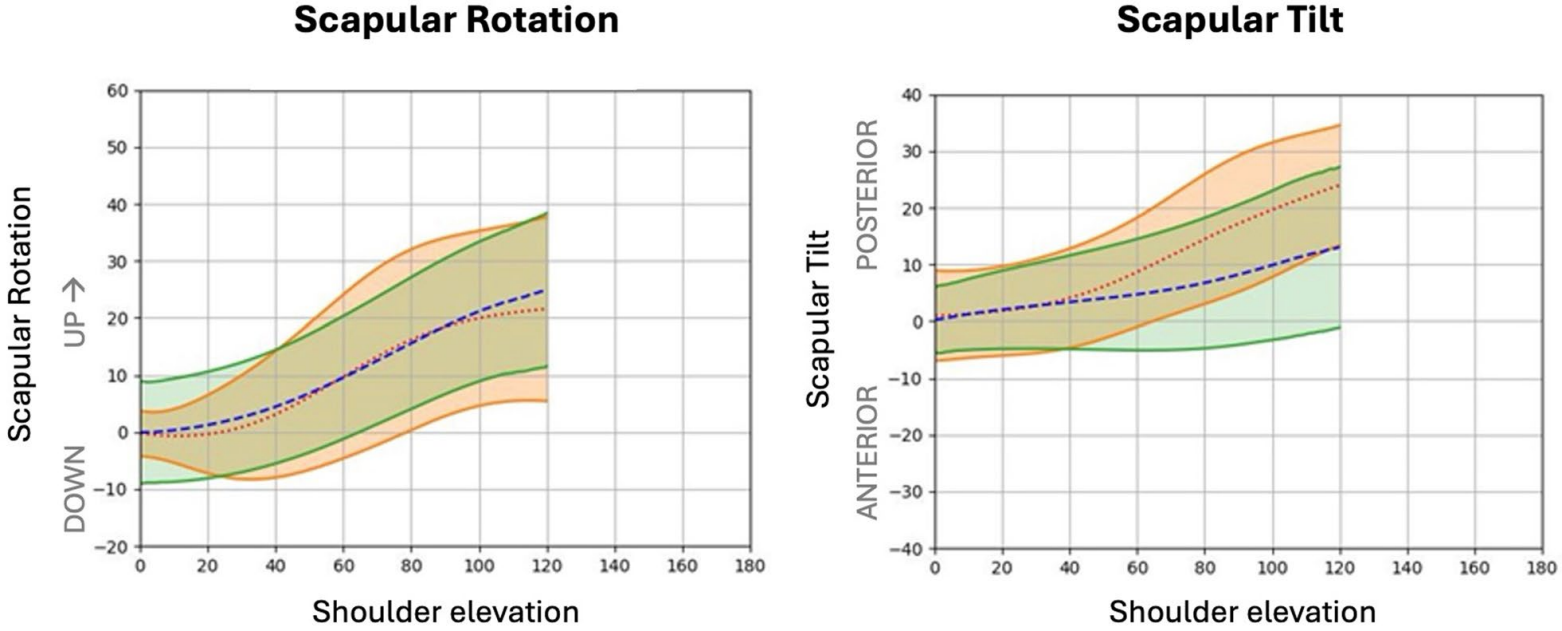
Scapular rhythm analysis during shoulder elevation. Graphics show the movement relationship between healthy (green area) and operated (orange area) shoulder. **A** Up and downward rotation; **B** scapular tilting

**Fig. 2:**
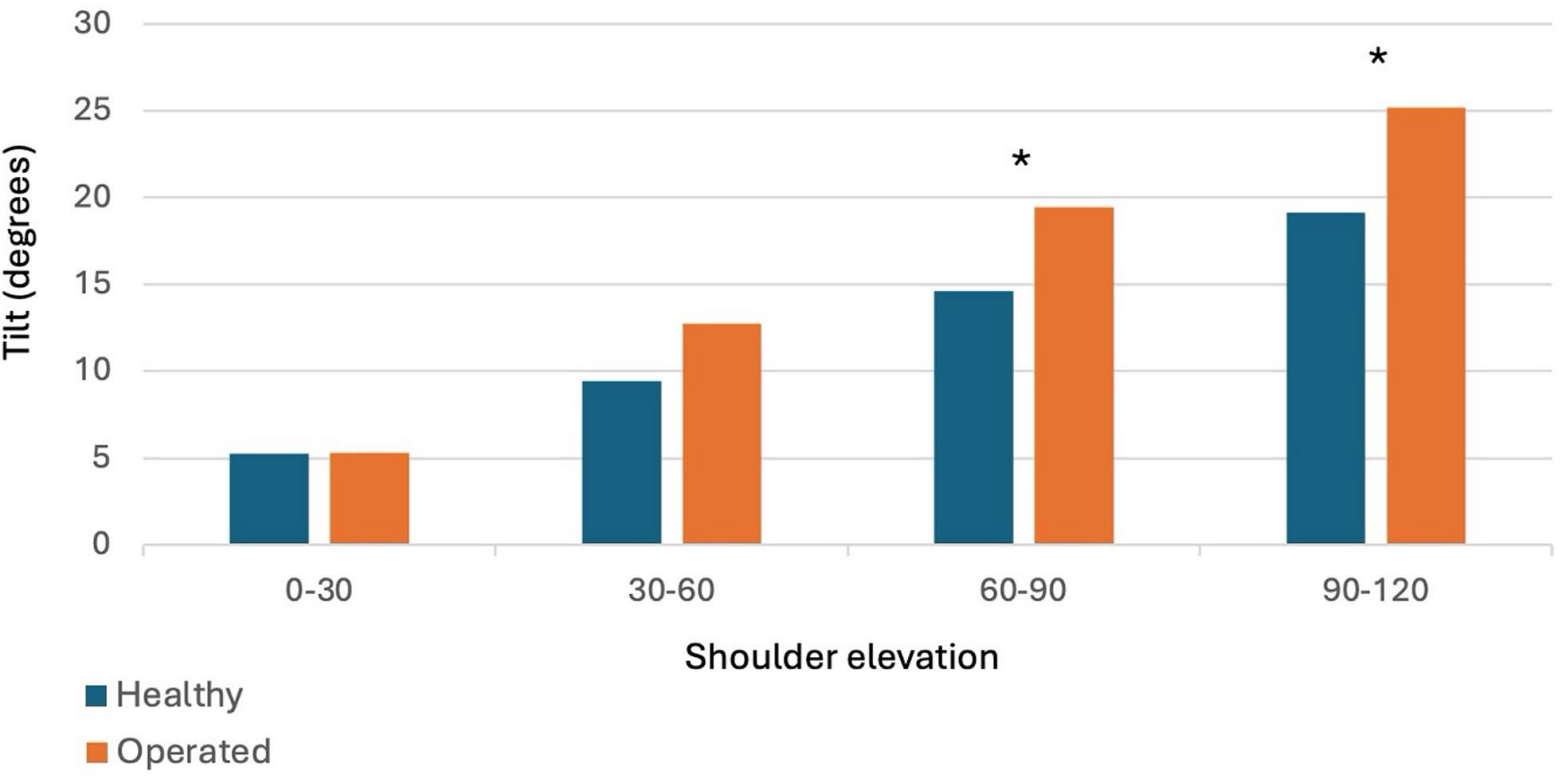
Mean of scapular tilting during shoulder elevation. *p < 0.05

Kinematic analysis revealed nearly normal scapulothoracic rhythm in all planes (Fig. 1A, B), with significant differences in scapular tilting during elevation and external rotation with adducted shoulder beyond 60° and 90° (*p* = 0.044 and *p* = 0.023, respectively), Fig. 2.

## Discussions

PMIRT is a disabling condition, and LDTT has been shown to be a viable treatment option when the subscapularis is functional and in absence of glenohumeral arthritis [2–5, 8, 12, 15]. Furthermore, this condition might contribute to scapulothoracic rhythm alterations [1]. Many studies have highlighted the importance of scapulohumeral kinematics in understanding shoulder function, reporting that up to 68% of patients with rotator cuff disease also present some form of scapular dyskinesis [13].

Following LDTT, the latissimus dorsi shifts from its natural role as an internal rotator and adductor to a potential external rotation and humeral depression (tenodesis) due to its new posterosuperior insertion. However, there is no consensus regarding to the effectiveness of its external rotation role. Neuromuscular training is critical for the success of this re-purposing.

Our data show that the operated shoulder might remain weaker than the contralateral side either in elevation and external rotation, but there is a significant improvement in external rotation strength when the LD is actively recruited in the transferred shoulder. EMG activity increased along with external rotation force, suggesting LD in its new transferred position actively acts during external rotation, not merely a tenodesis [2, 6].

While scapulothoracic rhythm was generally similar to the healthy shoulder, residual abnormalities in scapular tilting were observed, particularly increased posterior tilt at maximal forward elevation, which may reflect a compensatory mechanism contributing to range-of-motion recovery, and therefore warrants continued attention during rehabilitation.

Compared with prior work by Galasso et al., who evaluated scapular motion in elevation and abduction, our study adds data on shoulder function (strength and EMG activity) during external rotation [6, 10].

The clinical assessment showed similar range of movements with only a significant but slight difference in the active external rotation when comparing both shoulders. This suggests that LDTT is effective in restoring the range of motion. Although some suggest that LDTT is best suited for external rotation restoration at 90° of abduction rather than at neutral adduction, where lower trapezius transfer might be a better choice, our findings support its efficacy [9, 14].

This is probably due to our surgical strategy, which aims to achieve the maximum possible lever arm for humeral external rotation at neutral adduction. Furthermore, postoperative bracing in abduction and external rotation, along with a targeted muscle-strengthening rehabilitation program, may represent additional key factors contributing to the outcomes reported in our study. Rehabilitation protocols are essential, yet under-investigated [18].

One patient required RSA due to unsatisfactory results of LDTT. In this patient the subscapularis lesion was recognized only during the arthroscopic view, and then repaired with a suture anchor. It is widely accepted that good subscapularis function is essential for successful LDTT, as it maintains humeral head centering in both the horizontal and frontal planes [8].

This study has several limitations. First is its small sample size. The coronavirus disease 2019 (COVID-19) pandemic significantly impacted surgical activity, leading us to restrict patient selection to between July 2020 and June 2023. Earlier patients lacked proper physical therapy and follow-up. Additionally, the progressive resumption of surgical activity hindered patients’ recruitment. Second, since this is a retrospective study and preoperative clinical records were unavailable, we could not compare outcomes with preoperative condition. Third, while surface EMG provides valuable insights into muscle activation, its accuracy depends on electrode positioning and patient phenotype. Consequently, surface EMG does not provide a reproducible measurement between patients. That is why we opted to analyze the ratio of activity change rather than absolute muscle activity levels.

## Conclusions

LDTT improves shoulder function in patients with PMIRT, enhancing external rotation range of motion and strength when the LD is consciously activated, suggesting an active role in its transferred insertion.

LDTT results in a near-normal scapulothoracic rhythm postoperatively, with mayor residual tilting, highlighting the importance of incorporating scapular-focused therapy.

## Consent for publication

We authorize the author to proceed with the publication and, in this regard, we give our consent to move forward.

## Data Availability

All data and materials are available to the editor.

## Abbreviations

RSA: Reverse shoulder arthroplasty
LD: Latissimus dorsi
LDTT: Latissimus dorsi tendon transfer
MRI: Magnetic resonance imaging
RSA: Reverse shoulder arthroplasty
LHB: Long head biceps
ERR: External rotation force
ERADF: External rotation and adduction force
CMS: Constant-Murley score
ASES: Assessment Shoulder and Elbow Scale
QuickDASH: Quick Disability of the Arm, Shoulder and Hand
PMIRT: Posterosuperior massive irreparable rotator cuff tear
SR: Scapulothoracic rhythm
